# Acute Systemic Experimental Inflammation Does Not Reduce Human Odor Identification Performance

**DOI:** 10.1093/chemse/bjab004

**Published:** 2021-02-04

**Authors:** Arnaud Tognetti, Georgia Sarolidou, Julie Lasselin, Mats Lekander, Mats J Olsson, Johan N Lundström

**Affiliations:** 1 Department of Clinical Neuroscience, Division of Psychology, Karolinska Institutet, Stockholm, Sweden; 2 Department of Clinical Neuroscience, Osher Center for Integrative Medicine, Karolinska Institutet, Stockholm, Sweden; 3 Stress Research Institute, Department of Psychology, Stockholm University, Stockholm, Sweden; 4 Monell Chemical Senses Center, Philadelphia, PA, USA; 5 Department of Psychology, University of Pennsylvania, Philadelphia, PA, USA

**Keywords:** odor identification performance, lipopolysaccharide, interleukin-6 (IL-6), interleukin-8 (IL-8), tumor necrosis factor-α (TNF-α), MONEX-40

## Abstract

Olfactory dysfunction is a common symptom of various diseases, but the underlying pathophysiology has not been fully understood. Evidence from both animal and human studies suggests that local inflammation of the olfactory epithelium is linked to olfactory dysfunction. However, whether systemic inflammation causes olfactory dysfunction is yet to be determined. In the present behavioral study, we set out to test whether acute systemic inflammation impairs olfactory identification performance by inducing a transient and controlled state of systemic inflammation using an experimental endotoxemia model. We treated young healthy participants (*N* = 20) with a relatively high dose (2.0 ng/kg) of lipopolysaccharide (LPS) and a placebo treatment in a double-blind within-subject design, and assessed participants’ ability to identify odors using the MONEX-40, a reliable method for experimental assessment of odor identification ability in healthy and young individuals. Our results show that olfactory identification performance was not affected by the acute systemic inflammation triggered by the injection of LPS. Moreover, odor identification performance following the LPS injection was not associated with levels of circulating proinflammatory cytokines (interleukin-6, interleukin-8, and tumor necrosis factor-α). Because experimental LPS-induced systemic inflammation does not affect olfactory identification performance, our findings suggest that chronic, rather than transient, systemic inflammation is a more likely mechanism to explore in order to explain the olfactory deficits observed in inflammatory diseases.

## Introduction

Loss of smell (anosmia or hyposmia) is a symptom reflective of multiple chronic diseases (Klimek and Eggers 1997; Steinbach et al. 2011; Perricone et al. 2013; Aydın et al. 2016; Soler et al. 2020), but the underlying pathophysiology is not well understood. A proposed hypothesis is that olfactory dysfunction is linked to chronic inflammation of the olfactory epithelium (Lane et al. 2010; Wu et al. 2018) due to the neurotoxic effect of several proinflammatory cytokines that circulate locally in the sinonasal tissue (Vallieres et al. 2002; Kanekar et al. 2010; Li et al. 2013). Evidence from transgenic mouse models support this olfactory inflammation hypothesis with local overexpression of proinflammatory cytokines, such as tumor necrosis factor-α (TNF-α), resulting in inhibition of olfactory neuron function, turnover, and survival (Lane et al. 2010; Turner et al. 2010a, 2010b). Studies in human participants have further suggested that inflammation processes are involved in olfactory dysfunction. In fact, some evidence suggests that sensorineural olfactory loss may be the primary cause of hyposmia (clinically reduced sense of smell) in chronic rhinosinusitis patients (Konstantinidis et al. 2010; Yee et al. 2010). Indeed, chronic inflammation mediates a loss of functional olfactory neurons and triggers a replacement of the olfactory epithelium with respiratory epithelium (Yee et al. 2010). In addition, biopsies performed in the olfactory epithelium of patients diagnosed with chronic rhinosinusitis demonstrate higher concentrations of interleukin (IL)-5, IL-6, IL-8, and TNF-α compared with those from a control group (Lennard et al. 2000), and mucus cytokines levels (IL-5, IL-6, and TNF-α) in the olfactory cleft are negatively associated with olfactory dysfunction (Wu et al. 2018; Soler et al. 2020). Most compelling evidence of the olfactory inflammation hypothesis comes from clinical studies on patient diagnosed with chronic rhinosinusitis. However, a recent study also suggests that local inflammation plays a role in the acute olfactory loss described in patients with COVID-19 (Torabi et al. 2020). Taken together, there is strong evidence that local inflammation markers affect olfactory function. Notwithstanding the importance of the aforementioned findings, whether systemic inflammation causes olfactory dysfunction is still to be determined.

The idea that systemic inflammation may cause olfactory dysfunction is supported by several studies that found an association between disease severity and olfactory loss in patients diagnosed with psoriasis, granulomatosis with polyangiitis, and myasthenia gravis (Proft et al. 2014; Tekeli et al. 2015; Aydın et al. 2016). For example, a higher inflammatory activity, as measured by C-reactive protein serum levels (an inflammation marker that increases following IL-6 secretion), significantly correlates with a diminished olfactory function in patients diagnosed with granulomatosis with polyangiitis (Proft et al. 2014). However, various comorbidities are associated with such diseases. Whether systemic inflammation directly affects olfactory dysfunction is, thus, difficult to ascertain.

To the best of our knowledge, only 2 studies assessed whether systemic inflammation directly causes olfactory dysfunction in healthy participants. Henkin et al. (2013) showed that individuals diagnosed with olfactory hyposmia (*N* = 59), otherwise healthy, had significantly elevated levels of IL-6 in plasma compared with controls (*N* = 9). Nevertheless, in a more recent study using a large population-based sample of older adults (*N* = 1611), no significant relationship between odor identification performance and levels of circulating inflammation markers was found (Schubert et al. 2015), partly contradicting the former study. In addition, these studies have methodological problems that prevent a firm conclusion to be made. Both studies examined healthy participants with naturally low levels of circulating inflammatory markers for which the measures are sensitive to technical variations (e.g., fasting/nonfasting state, time of blood collection, storage temperature, sensitivity of the kits to measure cytokines). In addition, Henkin et al. (2013) only assessed a very small control sample (*N* = 9), whereas Schubert et al. (2015) assessed olfactory functions with a clinical cued odor identification test (SDOIT, Krantz et al. 2009) designed to detect anosmia and with a very low allowed variance (the test consists of only 8 items in total). More importantly, both studies used an observational, rather than an experimental, design making cause-and-effect relationships difficult to establish. Taken together, these methodological problems render a strong conclusion about a potential link between olfactory performance and immune responses difficult to be drawn.

In the present behavioral study, we set out to test the hypothesis that systemic inflammation reduces olfactory identification performance by inducing a controlled state of acute systemic inflammation using the model of experimental endotoxemia. This model has been extensively used in humans to study the effect of cytokines on brain functions and behavior (Schedlowski et al. 2014; Lasselin et al. 2020b). Here, we treated young, otherwise healthy, participants with a relatively high dose of a bacterial endotoxin (2 ng/kg body weight of lipopolysaccharide [LPS]) and a placebo treatment in a within-subject design, while measuring their ability to identify odors using the MONEX-40, a reliable odor ID test battery specifically designed to identify minor differences in identification ability between experimental conditions in healthy subjects (Freiherr et al. 2012). Based on the aforementioned literature, we hypothesized that during the LPS condition, participants would have poorer odor identification performance compared with the placebo condition, and that the decrease of olfactory identification performance induced by systemic inflammation would be negatively associated with participants’ levels of circulating proinflammatory cytokines (IL-6, IL-8, and TNF-α).

## Methods

The present study was part of a larger study aimed at assessing sickness detection, sickness behavior, and its predictors (Lasselin et al. 2017, 2018, 2020c; Regenbogen et al. 2017; Axelsson et al. 2018; Sarolidou et al. 2020). The study was approved by the Regional Ethical Committee of Stockholm (Dnr 2014/1946-31/1 and 2015/1415–32; ClinicalTrials.gov identifier: NCT02529592).

### LPS administration protocol

A more detailed protocol for the LPS administration that was used in this study can be found in Lasselin et al. (2017, 2018). In summary, 22 healthy participants (9 women, 13 men, mean age 23 years) were included in the protocol. The sample size was determined based on previous studies showing that 20–25 participants are sufficient to induce significant immunological and behavioral changes when using the model of experimental endotoxemia (Grigoleit et al. 2011; Calvano and Coyle 2012). The participants were recruited via advertisements at university campuses. Inclusion criteria included being between 18 and 50 years old, absence of physiological or psychiatric diseases, nonsmokers, nonexcessive alcohol users, and nonobese. All participants had a complete medical examination by a physician. Remuneration for participation was 3500 SEK. Informed written consent was obtained according to the Declaration of Helsinki from all participants prior to inclusion.

Participants were tested during 2 separate occasions in the Centre for Clinical Research at Danderyd Hospital, Stockholm, Sweden. They were randomly assigned to initially receive either a lipopolysaccharide injection (LPS; *Escherichia coli* endotoxin, Lot HOK354, CAT number 1235503, United States Pharmacopeia, Rockville, MD) at 2 ng/kg body weight concentration or a placebo injection (0.9% NaCl). Three to 4 weeks later (washout period), they received the reverse treatment. We used a double-blind experimental design: both participants and experimenters were unaware of the test condition. For safety reasons, participants’ health was monitored by a physician in both conditions. The physician responsible for the participants’ health was aware of the type of injection each participant received during the study day.

In the present study, 2 of the 22 initial participants were excluded (see below). The analyses were thus performed on 20 participants in total (8 women, 12 men, mean ± standard deviation = 23.05 ± 3.47 years old, range: 19–34 years).

### Proinflammatory markers assay

In both conditions (LPS and placebo), we collected blood samples before the injection and at 1, 1.5, 2, 3, 4, 5, and 7 h after the injection. Blood samples were analyzed to determine levels of proinflammatory cytokines (IL-6, IL-8, and TNF-α) using high-sensitivity multiplex (Human Mag Luminex Performance Assay, LHSCM000, LHSCM206, LHSCM208, and LHSCM210, RnD Systems, MN, USA). We used the peak values of each cytokine concentration after LPS administration, instead of a fixed time point (e.g., at the time of the odor identification task), to examine the relationship between olfactory identification performance and levels of cytokines. Indeed, the peak values represent the largest effect of LPS administration and reflect how much cytokines would have had an impact on identification performance (Lasselin et al. 2017, 2018). Data from one participant were not included in the analyses because of outlier IL-6 and IL-8 peak values (i.e., >3 S.D). Peak of IL-6 and IL-8 concentrations were observed 2 (*N* = 7 [35%], *N* = 11 [55%], respectively) and 3 h (*N* = 12 [60%], *N* = 9 [45%], respectively) after the LPS injection, except for one participant who exhibited IL-6 peak values at 1 h after injection. Peak of TNF-α concentrations was mostly observed 1.5 h after injection (*N* = 18 [90%]), the 2 other participants exhibiting peak values 2 h post-injection.

### Odor identification test

In both conditions (LPS and placebo), odor identification performance was assessed about 4 h 45 m after injection, using the 40-item Monell Extended Sniffin’ Sticks Identification Test (MONEX-40) (Freiherr et al. 2012). The test consists of 2 sets of 20 odor-filled felt-tip pens that each contains a unique odor. Participants were presented with the odor and a tablet screen displaying 4 names of alternatives associated with each odor pen. Their task was to identify the odor by picking one of the 4 labels. To assure that there was no effect of odor familiarization due to test retest, each set of 20-item odors was used to test odor identification performance in each condition (one participant was presented twice with the same set and was excluded from analyses) and counter balanced so that each set was used an equal amount of time in each condition. Previous data demonstrate that these 2 sets are matched with respect to the levels of difficulty (Freiherr et al. 2012).

### Statistical analyses

As indicated in our previous publication, we already have determined successfully experimental manipulation of the immune system by assessing change in body temperature and inflammatory markers between the LPS versus placebo condition (Lasselin et al. 2017, 2018). Nevertheless, we ensured that the experimental manipulation of the immune system was successful within our sub-sample of participants (*N* = 20 after the exclusion of 2 participants, see above). We compared the peak values of body temperature, and the peak concentrations of each proinflammatory marker, between the 2 conditions (LPS and Placebo) using paired samples *t*-tests. The peak concentrations of each proinflammatory marker were log-transformed to achieve normality. We also ensure that these measures still significantly differed between the 2 conditions 5 h after the injection, near the time of olfactory testing (there was a 15-min gap between the post 5-h blood sample collection and the odor identification task) using paired samples *t*-tests.

To examine whether an individual’s ability to identify the odors varied between the 2 conditions (LPS and placebo), we used a Bayesian generalized linear mixed-effects model (*bglmer* function in the *blme* R package) with a binomial error structure (the *bglmer* function applies a weak prior [Wishart] over the random effects to avoid singularity). Our dependent variable “Odor Identification” was an individual’s identification performance for each odor smelled (1 when he/she identified correctly the odor, 0 otherwise). “Inflammatory Condition” (LPS or placebo) was the explanatory variable. We included a random intercept for each participant’s (ParticipantID) and odor’s ID (OdorID) and following Barr et al. (2013), random slopes were specified maximally (for Inflammatory Condition by participant and by odor). In other words, the model was as follows: Odor Identification ~ Inflammatory Condition + (1 + Inflammatory Condition | ParticipantID) + (1 + Inflammatory Condition | OdorID).

We also examined whether the change in olfactory identification performance induced by the LPS injection was associated with the levels of circulating inflammation markers at peak. We analyzed our data using a Bayesian generalized linear mixed-effects model with a binomial error structure. We included random intercepts for each participant’s and odor’s ID, and random slopes for each cytokine concentration (IL-6, IL-8, and TNF-α) by odor’s ID. Our dependent variable “Odor Identification” was an individual’s identification performance for each of the 20 odors smelled during the LPS condition (1 when identified correctly the odor, 0 otherwise). The explanatory variables were the post-injection maximal values of each cytokine concentration. Following the recommendations found in Schielzeth (2010), we standardized each explanatory variable to compare the relative importance of the 3 markers with each other and to allow the model to converge. We also ruled out potential bias from multicollinearity as all explanatory variables demonstrated a low value of Variance Inflation Factor (all VIF< 2.2). Finally, we added in the model participants’ mean identification score of the placebo condition (“Baseline Performance”) to control for the interindividual variability of odor identification performance at baseline. The model was as follows: Odor Identification ~ IL-6 + IL-8 + TNF-α + Baseline Performance + (1 | ParticipantID) + (1+IL-6+IL-8+TNF-α | OdorID).

Statistical analyses were performed using R, version 3.6.0 (R Core Team 2017). The statistical significance of each variable was tested with likelihood ratio tests comparing the full model to those without the term of interest, and the α-level was set to 0.05.

## Results

### Experimental manipulation of the inflammatory response

**Figure 1.**
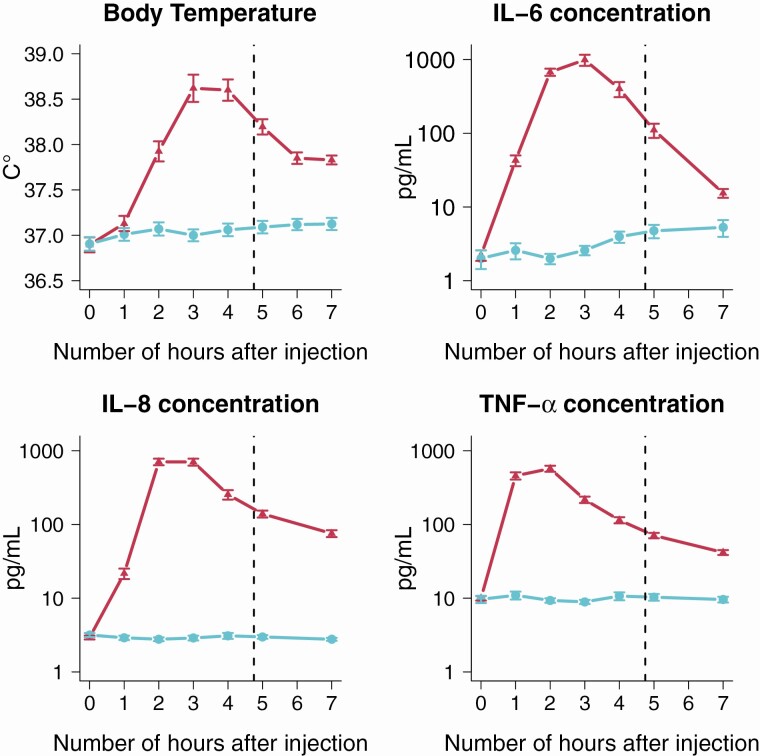
Effect of lipopolysaccharide (LPS) injection (red triangular-form dot) compared with placebo injection (blue dots) on participants’ body temperature and IL-6, IL-8, and TNF-α plasma levels (*N* = 20) during the 7 h following the injection. Error bars represent standard error of the mean. The odor identification test was performed at approximately 4 h 45 min after infection (dashed line), when the systemic inflammation was still present in LPS-treated participants but when the more severe effects had subsided.

During the LPS condition, participants (*N* = 20) demonstrated significantly higher peak of body temperature (paired samples *t*-test, *t*(19) = −12.92, *P* < 0.0001), as well as peak levels of IL-6 (*t*(19) = −24.08, *P* < 0.0001), IL-8 (*t*(19) = −35.92, *P* < 0.0001), and TNF-α (*t*(19) = −26.99, *P* < 0.0001) compared with the placebo condition. Five hours after the injection, when odor identification performance of the participants was assessed, participants still demonstrated significantly elevated body temperature (*t*(19) = −10.45, *P* < 0.0001) and levels of IL-6 (*t*(19) = −9.53, *P* < 0.0001), IL-8 (*t*(19) = −29.20, *P* < 0.0001), and TNF-α (*t*(19) = −13.66, *P* < 0.0001) in the LPS condition compared to the placebo condition (see Figure 1 for an illustration of the effect of the LPS administration over time).

### Effect of systemic inflammation on odor identification performance

**Figure 2.**
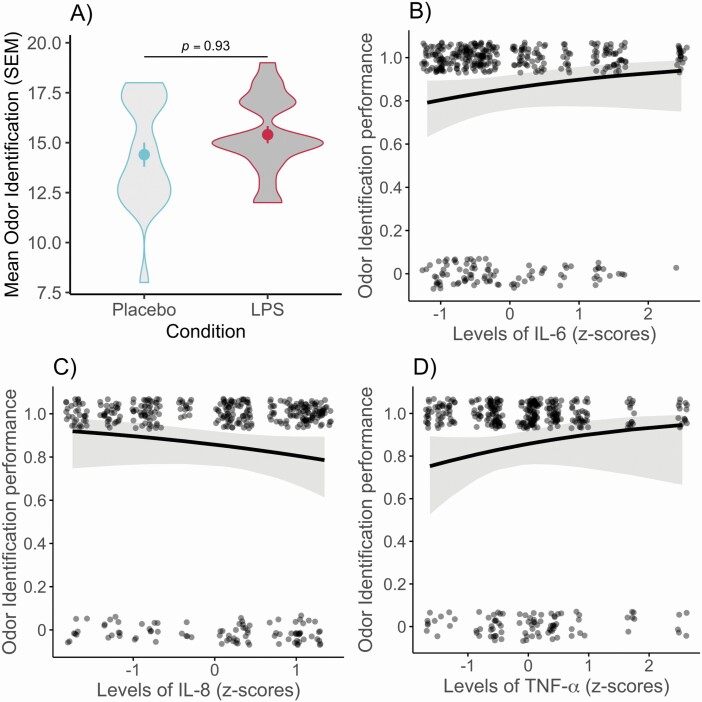
Odor identification during immune system activation. (**A**) Violin plot representing participants’ mean odor identification performance when the participants (*N* = 20) were exposed to either placebo injection or 2.0 ng/kg LPS injection. Error bars indicate standard error of the mean, and the violin plot outlines illustrate the distribution of the raw data. (**B–D**) Predicted probabilities (with 95% CI) of the association between mean odor identification performance and IL-6 (**B**), IL-8 (**C**), and TNF-α (**D**) levels during the LPS condition. Dots represent individual identification performance of each odor smelled (1 = correct identification, else 0) (*N* = 400).

Having determined that the experimental manipulation of immune system activation was successful with elevated immune markers following LPS administration, we then set out to determine whether the odor identification performance of the participants (*N* = 20) was affected. In contradiction to our hypothesis, condition (LPS or Placebo) did not affect participants’ olfactory identification performance (β = 0.03, SE = 0.30, χ  ^2^ (1, *N* = 800) = 0.01, *P* = 0.92; Figure 2A). Moreover, odor identification performance during the LPS condition was not associated with peak levels of either IL-6 (β = 0.38, SE = 0.28, χ  ^2^ (1, *N* = 400) = 1.87, *P* = 0.17; Figure 2B), IL-8 (β = −0.37, SE = 0.29, χ  ^2^ (1, *N* = 400) = 1.55, *P* = 0.21; Figure 2C), or TNF-α (β = 0.42, SE = 0.36, χ  ^2^ (1, *N* = 400) = 1.36, *P* = 0.24; Figure 2D). Similar results are obtained when keeping the participant who presented outlier levels of cytokines in the analyses: neither condition (β = 0.03, SE = 0.29, χ  ^2^ (1, *N* = 840) = 0.01, *P* = 0.93) nor peak levels of IL-6 (β = 0.81, SE = 0.51, χ  ^2^ (1, *N* = 420) = 2.52, *P* = 0.11), IL-8 (β = −0.91, SE = 0.57, χ  ^2^ (1, *N* = 420) = 2.51, *P* = 0.11) or TNF-α (β = 0.53, SE = 0.43, χ  ^2^ (1, *N* = 420) = 1.51, *P* = 0.22) were significantly associated with participants’ olfactory identification performance.

Among the 2 sets of 20-item odors that each participant smelled across both conditions, 3 of the 40 odor items exhibited identification levels in both conditions below 50%, which might indicate either unfamiliarity with the odor object or problems with the chemicals used to represent the odor object; a fact that might obscure potential effects. To assure that this did not modulate the negative results reported above, we excluded these 3 items from the overall odor identification performance for both conditions. However, analyses without these 3 odor items still did not show any association between odor identification performance and inflammatory condition (β = 0.02, SE = 0.31, χ  ^2^ (1, *N* = 740) = 0.01, *P* = 0.96) or with IL-6 (β = 0.45, SE = 0.32, χ  ^2^ (1, *N* = 370) = 1.98, *P* = 0.16), IL-8 (β = −0.42, SE = 0.34, χ  ^2^ (1, *N* = 370) = 1.54, *P* = 0.21), or TNF-α (β = 0.52, SE = 0.41, χ  ^2^ (1, *N* = 370) = 1.57, *P* = 0.21) peak levels during the LPS condition.

## Discussion

The goal of the present behavioral study was to examine the effect of systemic inflammation on olfactory identification performance in healthy participants. Our results showed that olfactory identification performance was not affected by the acute systemic inflammation triggered by the injection of LPS. Moreover, no associations were found between the change of olfactory performance followed by LPS injection and levels of circulating proinflammatory cytokines. Hence, our findings suggest that experimental endotoxemia does not affect olfactory identification performance.

As noted, olfactory dysfunction is a common symptom of various diseases, ranging from COVID-19 (Cazzolla et al. 2020; Torabi et al. 2020) to autoimmune/immune-mediated (Perricone et al. 2013) or neurodegenerative diseases (Ponsen et al. 2009). For example, both chronic rhinosinusitis and allergy rhinitis are strongly associated with a decrease in odor threshold and identification performance (Klimek and Eggers 1997; Soler et al. 2016). It has been suggested that the olfactory dysfunction observed in these diseases may be caused by local and systemic inflammation. Although there is compelling evidence attesting the negative effect of local inflammation on olfactory functions (Lennard et al. 2000; Lane et al. 2010; Turner et al. 2010a, 2010b; Yee et al. 2010; Wu et al. 2018), evidence demonstrating whether systemic inflammation causes olfactory dysfunctions remains scarce. Specifically, several studies found a correlation between olfactory dysfunction and levels of circulating inflammatory markers in patients diagnosed with chronic rhinosinusitis, granulomatosis with polyangiitis, or COVID-19 (Wu et al. 2018; Soler et al. 2020; Torabi et al. 2020). In addition, a previous study has shown that patients with a diagnosed olfactory hyposmia, but that were otherwise healthy, had significantly elevated levels of IL-6 in plasma and nasal mucus compared with healthy controls, which suggests a direct link between levels of proinflammatory markers and olfactory dysfunction (Henkin et al. 2013; see also Schubert et al. 2015). However, these aforementioned studies used an observational approach that does not enable to ascertain whether systemic inflammation directly affects olfactory loss. Building on these findings, we hypothesized that an experimental systemic inflammation would affect olfactory identification performance. However, our participants’ odor identification performance was not disturbed while experiencing an acute systemic immune response, as evidenced by a transient increase in circulating proinflammatory cytokines. One possible explanation for the absence of any effect on olfactory performance could be that our experimental disease model rendered our participants sick only for a few hours and that a longer period of systemic inflammation is necessary to result in measurable olfactory perturbations. On the other hand, the level of circulating IL-6 experienced by the participants at the peak of inflammation (2 h after the LPS injection) was 1000 times higher, on average, compared with the level measured in patients suffering from hyposmia in a previous study (Henkin et al. 2013), and it was still 100 higher 5 h after injection, when the olfactory identification performance of participants was assessed. Thus, it seems unlikely that the level of the inflammatory response, per se, induced by the LPS administration was not high enough to affect olfactory identification performance.

The present study is the first, to our knowledge, to assess olfactory performance in an experimental endotoxemia model using a reliable method to assess odor identification abilities. Nevertheless, this study is also subject to some limitations. First, experimental procedures involving human interventions do not allow testing a large number of participants. Indeed, strict ethical guidelines regulate these experimental procedures such as restricting the number of participants to the minimal sample size allowing small effect sizes to be detected. Based on previous published papers that reported a very strong effect of experimental endotoxemia on behavior, we calculated that a sample size of 20 participants was large enough to detect small effect sizes (Grigoleit et al. 2011; Calvano and Coyle 2012). It needs also to be mentioned that our sample size was similar to or higher than several previous naturalistic studies that found a significant link between the levels of proinflammatory markers in plasma or nasal mucus and olfactory loss (Lennard et al. 2000; Henkin et al. 2013). Second, we tested participants’ ability to identify odors as a proxy for olfactory function. This is an approach that has been repeatedly demonstrated to be a good estimate of general olfactory function (Doty et al. 1984) and is commonly used in both experimental (Seubert et al. 2013) and clinical testing (Hummel et al. 2007). However, even though tests of various olfactory subfunctions, such as ability to discriminate between odor qualities, odor detection threshold, or cued odor identification, demonstrate high intercorrelations (Doty et al. 1994), cued odor identification tasks are only partly dependent on odor acuity. To at least some degree, performance on an odor identification test is dependent on nonolfactory cognitive factors such as language skills and object recognition and comprehension (Dulay et al. 2008). Hence, in spite of the fact that we are unable to demonstrate an association in this study, it is possible that there are associations between olfactory dysfunction and immune system responses that would be observed if a more extensive odor detection threshold test had been used. Such a test is a more direct estimate of olfactory acuity (Hedner et al. 2010). However, it would have, also, been more laborious and more prone to fatigue-related effects that would potentially bias the results to demonstrate decreased performance during the LPS condition where participants are more fatigued due to their experimental sickness state (Lasselin et al. 2017, 2020a). These potential problems notwithstanding, future studies should, if the experimental manipulation allows, use tests of detection threshold to more directly assess links between olfactory acuity and immune responses. Another interesting idea for future studies is the possibility that systemic inflammation could influence olfactory identification performance differently in the short term versus long term. Indeed, identification performance is prone to cognitive influences. In the short term, systemic inflammation may thus affect positively cognition on the perception of odors as a reinforcement of the protective function of olfaction (danger detection). In other words, systemic inflammatory activation could trigger a transient state of vigilance, leading to increased allocation of cognitive resources to the processing of olfactory information, especially those related to odors signaling a threat. Contrary to our prediction, systemic inflammation could thus increase, in the short term, olfactory identification performance for at least some type of odors that would need to be avoided (e.g., contaminated food).

To conclude, this study reports that odor identification is resistant to an acute increase in systemic inflammation, which thus suggests that chronic, rather than transient, systemic inflammation is a more likely mechanism to explore in order to explain the olfactory deficits observed in inflammatory diseases.
